# Correspondence

**Published:** 1897-03

**Authors:** Burton Leonard

**Affiliations:** Tampa, Fla.


### Correspondence.

---

EDITOR SOUTHERN MEDICAL RECORD:

SIR—Some time ago an individual of the genus chiffonier, and who for lucidity's sake, I will call Garibaldi, came to me with a dose.

Although he had not been in the States long enough to speak American in all its purity, yet his period of residence was sufficient for him to catch on to the prehistoric castanea of straining himself. Seeing that I was skeptical as to his friend getting "inflamma," as he called it, in that way, he searched back through his memory and found the woman "onea yalla galla." Things never went right from the beginning of the treatment; three-quarters of the time I could not understand him, and four quarters of the time he could not understand me, and there was trouble, I avow. To make matters worse, he had everlasting faith in local synergists, such as having an old negress "crack his back;" trying to whirl it off at the Congo dances to the music of tom-toms and ki-yis, and drinking great quaffs of a decoction of the pound cake bush. As he was paying me in city scrip, which was a long way from par, I decided to wind it up one way or the other, and ordered an injection of nitrate of silver, strong enough to knock a gonococcus silly in one round.

The next morning when I came back to my office after my usual rounds, I found him waiting. "*Bien*," I said, "*comment va la chande-pisse et l'injection neuve*." Jumping up, hands aloft, the gleaming eyes and teeth and a wild Mafia expression over the rest of his face, brought back in a flash the remembrance of my seeing "Cavalleria Rusticana" at De Give's one night while I was a student at the Southern.

By the bye, I believe my friend the Israelitish Hebrew Jew Diamond was "*a la pistache*," too.

The gentleman in the black smoking-cap who manipulated the bass violin bent low over his instrument, there was a moment's pause, a boyish voice cried "Beerman and Silverman's fine candies, twenty-five cents a package," then a scream from the orchestra and above the awful melody was the voice of the rag-picker, "Doctor, God damma, burna lika hella."

BURTON LEONARD, M. D.

*Tampa, Fla.*

